# Diversity and Community Structure of Soil Bacteria of Different Vegetation Types in Volcanic Lava of Wudalianchi, China

**DOI:** 10.3390/microorganisms14030666

**Published:** 2026-03-15

**Authors:** Jiahui Cheng, Lihong Xie, Mingyue Jiang, Hongjie Cao, Fan Yang, Qingyang Huang

**Affiliations:** 1 Institute of Natural Resources and Ecology, Heilongjiang Academy of Sciences, Harbin 150040, China; chengjiahui407@163.com (J.C.); xielihong903@163.com (L.X.); 13804585147@163.com (M.J.); hjcao781228@163.com (H.C.); 2 National and Provincial Joint Engineering Laboratory of Wetlands and Ecological Conservation, Harbin 150040, China

**Keywords:** bacteria, volcanic lava, microbial communities, vegetation types, Wudalianchi

## Abstract

Volcanic lava has a complete primary succession; the plant community composition can explain a great part of the variation of soil microbial diversity and community structure. Bacteria dominate the soil microbial communities in abundance and diversity, and they are important drivers of organic matter decomposition and nutrient cycling. With 16S rRNA Illumina Miseq sequencing techniques, we analyzed the soil bacterial communities and diversities associated with different vegetation types in Wudalianchi. Shrub soils had the highest pH, MC, TOC, TN, AP, AN and NN, whereas moss soils had the lowest. The Shannon, Ace, and Pd indices of bacteria showed significant differences in the different vegetation types (*p* < 0.05). Bacterial Ace, Shannon, and Simpson indices peaked in Herb and Shrub is highest. The Proteobacteria, Actinobacteriota, Acidobacteriota, Planctomycetota and Chloroflexota were the most abundant groups at phyla level. Bacterial community composition varied significantly across vegetation types (*p* < 0.05). At the family level, *Pseudonocardiaceae* predominated in moss soils. Redundancy analysis and correlation analysis revealed MC, pH, and TP as key environmental factors shaping bacterial communities. Functional predictions based on taxonomic data indicated that chemoheterotrophy and aerobic chemoheterotrophy were the predominant functional groups. In conclusion, although soil microbial composition and diversity differed markedly across vegetation types following volcanic eruptions, functional groups prioritized carbon fixation strategies.

## 1. Introduction

Soil microorganisms serve as the cornerstone of terrestrial ecosystems, playing a crucial role in monitoring ecosystem succession and soil dynamics [1,2]. Among soil microorganisms, bacteria represent the most abundant and diverse group [3,4], actively participating in organic matter decomposition, nutrient cycling, and various essential soil processes [5,6]. Moreover, bacteria exhibit high sensitivity to even minor changes in the soil environment, eliciting significant responses [7,8]. Vegetation shapes soil bacterial communities both directly and indirectly by altering soil physicochemical properties [9]. Soil bacterial communities differ markedly across forest types—including broad-leaved forests, coniferous and mixed coniferous-broadleaf forests [10]. These differences are primarily driven by vegetation-induced changes in soil properties. Different vegetation types regulate soil pH through root exudates and nitrogen uptake preferences of dominant plants, directly shaping distinct bacterial communities adapted to local conditions [11], while differences in the quality and composition of their litter alter key soil attributes consequently influencing bacterial community composition and function [12].

Volcanic eruptions serve as a classic starting point for primary succession, providing a natural laboratory for investigating the dynamic changes in soil microbial communities during vegetation succession. Volcanic ash formed after volcanic eruptions promotes the decomposition of soil organic matter, releases nutrients, enhances plant nutrient availability, and facilitates tree growth [13]. Meanwhile, volcanic soils influence soil shape microbial composition, enrich beneficial bacterial taxa, and enhance the soil microecological environment and improve the soil microecological environment [14]. Volcanic sediments can indirectly affect nutrient cycling in soils by influencing the availability of organic mineral content through bacterial activity [15]. Therefore, studying soil bacteria in different vegetation types within volcanic forests holds significant importance for elucidating the structure and function of extreme ecosystems, optimizing vegetation restoration, and developing microbial resources.

The Laoheishan Volcano in Wudalianchi (WDLC) volcano erupted between 1719 and 1721, resulting in the formation of a 65 km^2^ lava plateau [16]. Volcanic eruptions can lead to significant landscape changes, causing disturbances that greatly impact soil, vegetation dynamics, and ecosystem function [17]. Following the eruption, primary succession commences on bare rock, with microorganisms being among the initial colonizers of the newly exposed volcanic substrates [18,19]. In recent years, research on vegetation ecology in the Wudalianchi volcanic area has increasingly focused on topics such as the adaptability of dominant volcanic plants, soil microbial biomass and diversity, and the interactions between vegetation and soil [20,21,22]. However, it remains unclear how different vegetation types influence soil bacterial community structure and diversity in the context of volcanic lava, and how these changes affect the ecological functions of microbial communities. In this study, we analyzed soil bacterial community composition and potential functions under five common vegetation types in the Wudalianchi volcanic area—moss, herb, shrub, broad-leaved forest, and coniferous-broadleaf mixed forest. We investigated the differences in soil nutrients, microbial community composition, and functional profiles among these vegetation types, aiming to provide insights into the ecological impacts of different vegetation types on volcanic lava platforms.

## 2. Materials and Methods

### 2.1. Location Description and Soil Sampling

**Figure 1:**
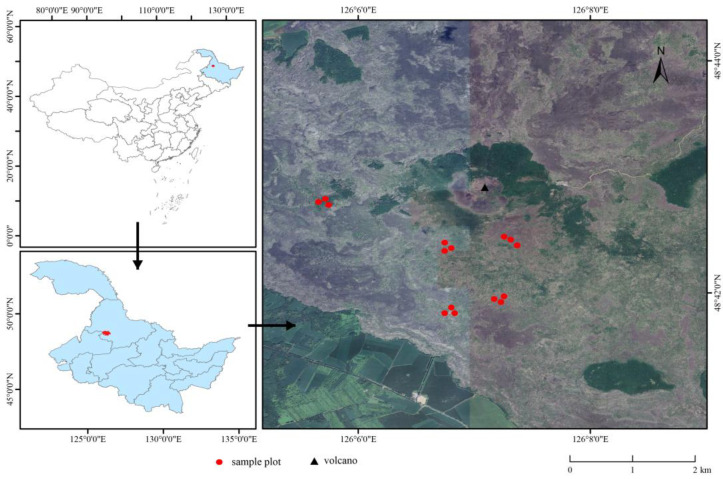
Spatial distribution of sampling locations across the study area.

The WDLC (125°45′–126°30′ E; 48°30′–48°50′ N) is situated in the northwestern region of Heilongjiang Province, China. The area experiences a continental monsoon climate characterized by harsh, lengthy winters and mild, brief summers. The mean annual temperature in the region is −5 °C, with annual precipitation averaging 476.33 mm. The dominant soil type in the WDLC region is volcanic ash soil (Figure 1) [23].

The most recent volcanic eruption took place at Laoheishan between 1719 and 1721, resulting in the formation of a 65 km^2^. Following this event, the eruption at Laoheishan eradicated the existing vegetation and soil, leading to the initiation of primary succession on bare rock. This process encompassed various stages of plant succession, including mosses, herbaceous plants, shrubs, broad-leaved forests, coniferous and mixed broad-leaved forests.

In August 2023, following a volcanic eruption, three sites were selected to collect surface soil samples (0–5 cm depth) from different vegetation types, including moss, herb, shrub, broadleaf forest, and coniferous and broad-leaved mixed forests (Table 1). The surface soil layer (0–5 cm) represents the most active horizon during post-volcanic ecosystem recovery, characterized by rapid organic matter accumulation, high microbial activity, and the greatest sensitivity to vegetation succession and environmental factors. Sampling locations were carefully chosen to ensure consistent elevation and soil type across all sites. At each vegetation type within a site, sampling was conducted in three 10 m × 10 m quadrats positioned approximately 20 m apart. Sampling procedures avoided tree trunks and plot edges. After clearing surface litter and removing stones and roots, soil samples were taken from the 0–5 cm depth following a five-point sampling pattern. Within each quadrat, three subsamples were taken and homogenized to form a composite sample weighing approximately 1000 g. These composites were brought to the laboratory, where they were sieved through a 2 mm mesh to remove roots and plant debris. Each sieved sample was then divided: one portion was stored at −80 °C for DNA extraction and microbiological analysis, with the remaining sample designated for analyzing soil physical and chemical attributes.

### 2.2. Determination of Soil Physical and Chemical Factors

We measured soil pH by preparing a 1:5 soil-water suspension and using a pH meter [24]. Soil moisture content (MC) was measured by oven-drying duplicate subsamples at 105 °C overnight. The total organic carbon (TOC) and total nitrogen (TN) were determined with an elemental analyzer (EA3000, Euro Vector, Foggia, Italy) [25]. The total phosphorus (TP) was determined using molybdenum antimony colorimetry [26]. The available phosphorus (AP) was determined using NaHCO_3_ extraction colorimetry [27]. Ammonium nitrogen (AN) and nitrate nitrogen (NN) were determined by a potassium chloride-flow analyzer (Skalar, Breda, The Netherlands) [28]. All soil physicochemical analyses were performed with three analytical replicates per sample.

### 2.3. Determination of Soil Bacteria

We extracted bacterial genomic DNA using the Power Soil DNA Isolation Kit (MoBio, Carlsbad, CA, USA) as described in the manufacturer’s guidelines. We amplified the V3–V4 regions of the 16S rDNA gene from soil bacteria using barcoded primers 515F (5′-GTFYCAGCMGCCG CGGTAA-3′) and 806R (5′-GGACTACNVGGGTWTCTAAT-3′). PCR amplification was performed under the following conditions: initial denaturation at 95 °C for 3 min, followed by 30 cycles of denaturation at 95 °C for 30 s, annealing at 55 °C for 30 s, extension at 72 °C for 45 s, and a final extension at 72 °C for 10 min. PCR products were purified and sequenced on the Illumina MiSeq PE300 platform (Illumina, San Diego, CA, USA) using paired-end sequencing by Shanghai Majorbio Bio-Pharm Technology Co., Ltd. (Shanghai, China).

Raw sequencing reads were demultiplexed and quality-filtered using QIIME2 (version 2020.11). The DADA2 plugin was employed for denoising, including read merging, quality filtering (removing reads with an average quality score < 20), and chimera removal, resulting in amplicon sequence variants (ASVs). Taxonomic assignment of ASVs was performed against the SILVA 138/16S rRNA database at a 99% confidence threshold. Due to the limitations of 16S amplicon sequencing, taxonomic classification was performed at the phylum and family levels, and species-level identification was not considered. We conducted all bioinformatics analyses via the Majorbio Cloud Platform (www.majorbio.com).

### 2.4. Statistical Analysis

The mean and standard error of soil physical and chemical factors were calculated. We employed one-way ANOVA with Duncan’s post hoc test (α = 0.05) to compare soil physicochemical properties, bacterial community composition, and alpha diversity index across vegetation types. All statistical analyses were conducted using SPSS 20 for Windows.

Alpha diversity indices (Sobs, Ace, Shannon and Pd) were determined based on ASV data to evaluate species richness and diversity within soil bacterial communities [29]. Beta diversity was visualized by principal coordinate analysis (PCoA) of Bray–Curtis dissimilarity matrices using the vegan package (version 2.5-7) in R software (version 3.5.1, R Core Team, 2018). To statistically evaluate differences in bacterial community structure across vegetation types, analysis of similarities (ANOSIM) and permutational multivariate analysis of variance (PERMANOVA) were performed on the same distance matrices, each with 999 permutations implemented in the vegan package.

The relationships between soil biochemical properties (pH, MC, TOC, TN, TP, AP, AN, NN) and microbial community composition were assessed using Pearson correlation analysis. Prior to redundancy analysis (RDA), soil factors were assessed for multicollinearity using the variance inflation factor (VIF), and variables with VIF > 10 were excluded. RDA was employed to explore the associations between selected soil factors (pH, MC, TP, AP) and the composition of soil bacterial communities. RDA analyses were performed in R (version 3.5.1) with the vegan package (version 2.5-7), and two-dimensional ordination plots were generated for visualization. Functional prediction of bacterial communities was performed using FAPROTAX (version 1.2.1) to examine the potential ecological roles of soil bacteria based on the ASV table.

## 3. Results

### 3.1. Soil Physical and Chemical Properties

The soil physical and chemical characteristics were significantly different among the five vegetation types (Table 2). Shrub (S) exhibited the highest pH values and concentrations of MC, TOC, TN, AP, AN, and NN, whereas moss (M) areas displayed the lowest levels of these parameters. However, moss (M) areas had the highest concentration of TP. This study employed single-time sampling, and the results represent only the conditions in August 2023.

### 3.2. Soil Bacterial Diversity

After sequencing and post-processing, 940,583 informative sequences were obtained, including 62,705 bacterial sequences, with an average length of 376 base pairs. Rarefaction curves tended toward saturation as sequencing depth increased, indicating that the sequencing coverage was adequate to represent the majority of soil microbial communities. Variations in alpha diversity indexes (Ace index, Shannon index, Coverage index, Simpson evenness index, and Pd index) were observed across various vegetation types (Table 3). The highest values for Ace, Shannon, and Simpson indices among soil bacteria were recorded in habitats H and S.

**Figure 2:**
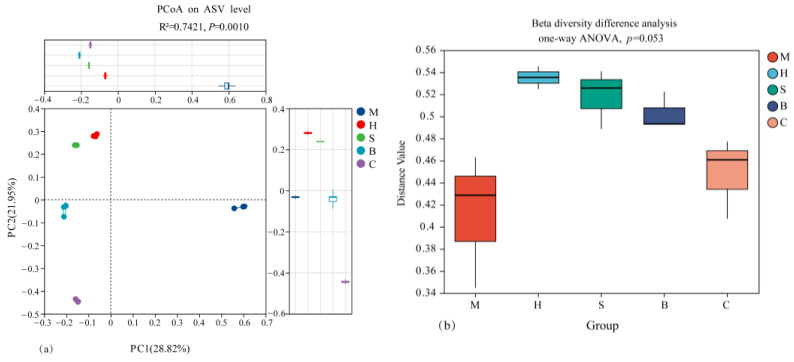
Principal coordinates analysis and *Beta* diversity difference analysis of bacterial community structure. M: moss; H: herb; S: shrub; B: broadleaf forest; C: coniferous and broad-leaved mixed forest. (**a**) PCoA plot showing the distribution of bacterial communities across different vegetation types. (**b**) Beta diversity difference analysis (one-way ANOVA) showing distance values for each vegetation type.

PCoA showed significant differences in soil bacteria in different vegetation types at the ASV level, PCoA1 accounted for 28.82% of the total variance, and PCoA2 accounted for 21.95% (Figure 2a). The bacterial community within the same group was clustered, while different vegetation communities were separated. The bacterial community of M showed significant differences among vegetation types (Figure 2b).

### 3.3. Bacterial Community Composition

**Figure 3:**
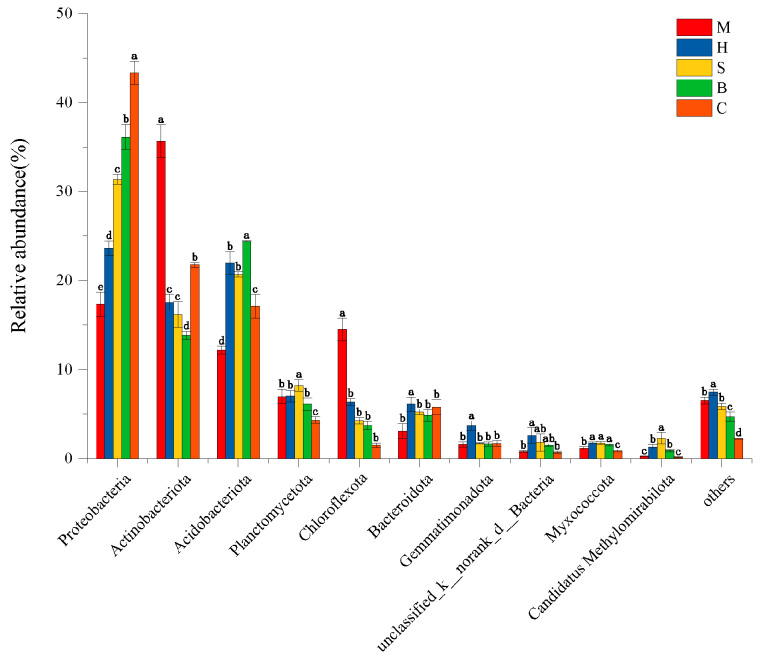
Relative abundance of bacterial community structure in different vegetation types (M: moss; H: herb; S: shrub; B: broadleaf forest; C: coniferous and broad-leaved mixed forest) at the phylum level. Significant differences (*p* < 0.05) are indicated by different letters, while the same letter indicates no significant difference.

Eleven bacterial phyla showed relative abundances above 1% at the phylum level (Figure 3). The Proteobacteria (17.33–43.33%), Actinobacteriota (13.86–35.65%), Acidobacteriota (17.12–24.40%), Planctomycetota (4.32–7.02%) and Chloroflexota (1.50–14.50%) were the most abundant group. Proteobacteria was the most abundant phylum in C. Its relative abundance also increased significantly as plant succession progressed (*p* < 0.05). Actinobacteriota and Chloroflexota were the most abundant groups in M, Acidobacteriota was the most abundant group in B and Planctomycetota was the most abundant group in S.

**Figure 4:**
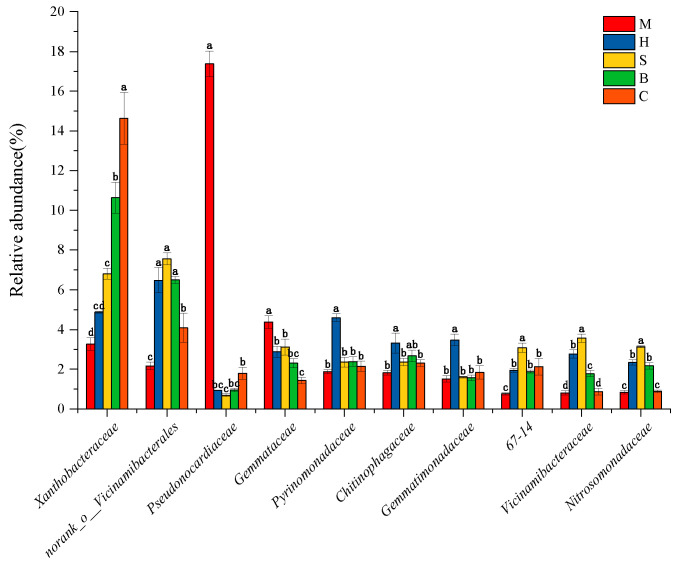
Relative abundance of bacterial community structure in different vegetation types (M: moss; H: herb; S: shrub; B: broadleaf forest; C: coniferous and broad-leaved mixed forest) at the family level. Significant differences (*p* < 0.05) are indicated by different letters, while the same letter indicates no significant difference.

Analysis revealed significant heterogeneity in family-level community composition: *Pseudonocardiaceae* was the dominant group in M, *Xanthobacteraceae* was the dominant group in B and C, *norank_o__Vicinamibacterales* was the dominant group in H and S (Figure 4).

### 3.4. RDA

**Figure 5:**
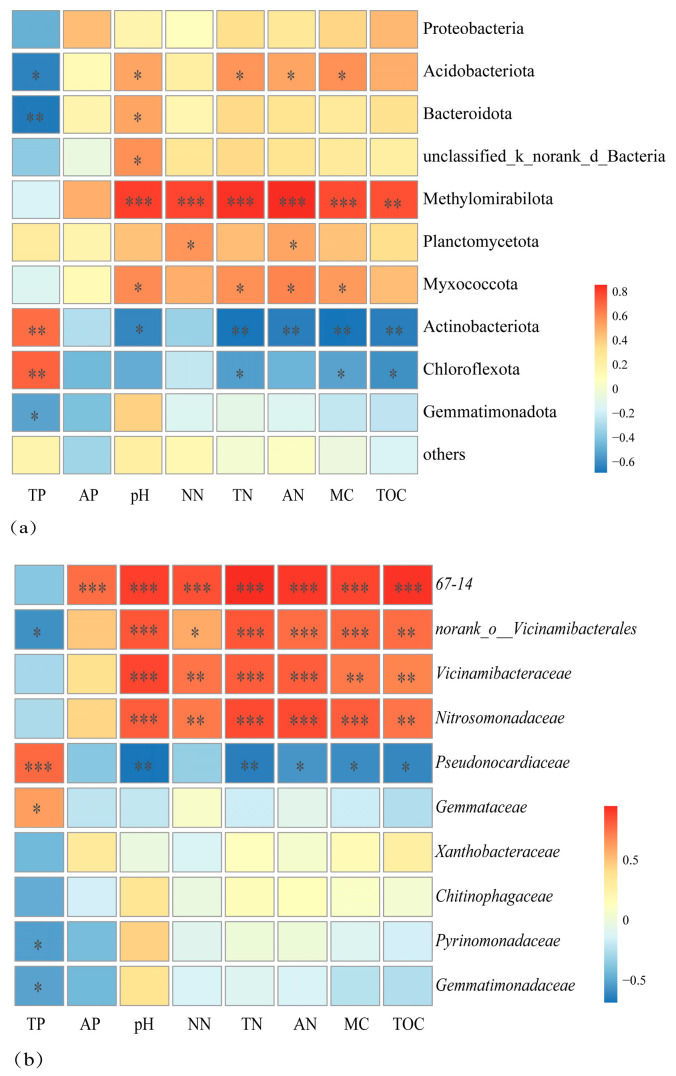
Heatmap of Pearson’s correlation coefficients between soil characteristics and microbial community composition at the phylum (**a**) and family (**b**) levels. Significance thresholds: *** *p* < 0.001, ** *p* < 0.01 and * *p* < 0.05.

Pearson correlation analysis showed that soil bacterial community Candidatus Methylomirabilota [30], Acidobacteriota and Myxococcota were significantly positively correlated with soil pH, TN, AN, and MC (*p* < 0.05, Figure 5a). Actinobacteriota and Chloroflexota correlated negatively with TN, MC, and TOC, but positively with TP (*p* < 0.05).

At the family level, soil pH, NN, TN, AN, MC, and TOC were significantly positively correlated with *67-14*, *norank_o_Vicinamibacterales*, *Vicinamibacteraceae*, and *Nitrosomonadaceae* (*p* < 0.05, Figure 5b). By contrast, these soil variables were negatively correlated with *Pseudonocardiaceae*, whereas TP showed a positive correlation with this family (*p* < 0.05).

**Figure 6:**
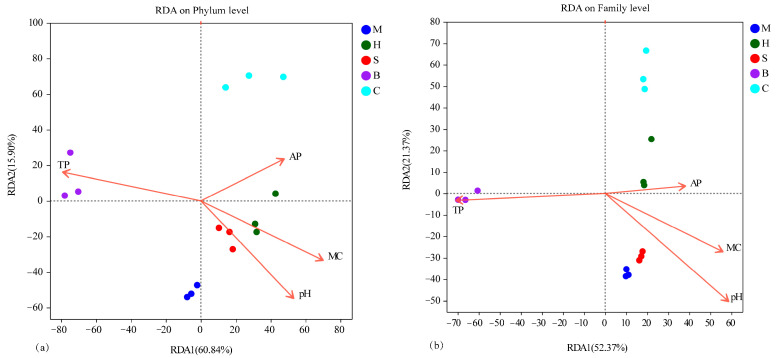
Redundancy analysis (RDA) ordination of soil bacterial communities constrained by soil physicochemical properties at the phylum (**a**) and family (**b**) levels. M: moss; H: herb; S: shrub; B: broadleaf forest; C: coniferous and broad-leaved mixed forest.

The RDA was performed on bacterial communities among different vegetation types at the phylum (Figure 6a) and family (Figure 6b) level. Soil factors were assessed for multicollinearity using the variance inflation factor (VIF), with moderate values observed for MC (VIF = 3.22), pH (VIF = 2.88), AP (VIF = 2.86), and TP (VIF = 1.70). The first two RDA dimensions captured 60.84% and 15.90% of the variance in bacterial composition at the phylum level. Total phosphorus (TP) (R^2^ = 0.472, *p* = 0.022), pH (R^2^ = 0.446, *p* = 0.036), and MC (R^2^ = 0.451, *p* = 0.041) significantly influenced the bacterial community composition. For family-level assemblages, RDA revealed that the first two axes accounted for 52.37% and 21.37% of the total variability. The driving factors that significantly affected the bacterial community structure were pH (R^2^ = 0.741, *p* = 0.001), TP (R^2^ = 0.576, *p* = 0.007), and MC (R^2^ = 0.472, *p* = 0.023). The samples were clearly separated into different vegetation types, and the bacteria were distinct among vegetation types. The combined results of VIF and RDA highlighted pH, TP, and MC as the primary variables influencing the bacterial community structure.

### 3.5. Bacterial Function Prediction in Soil

**Figure 7:**
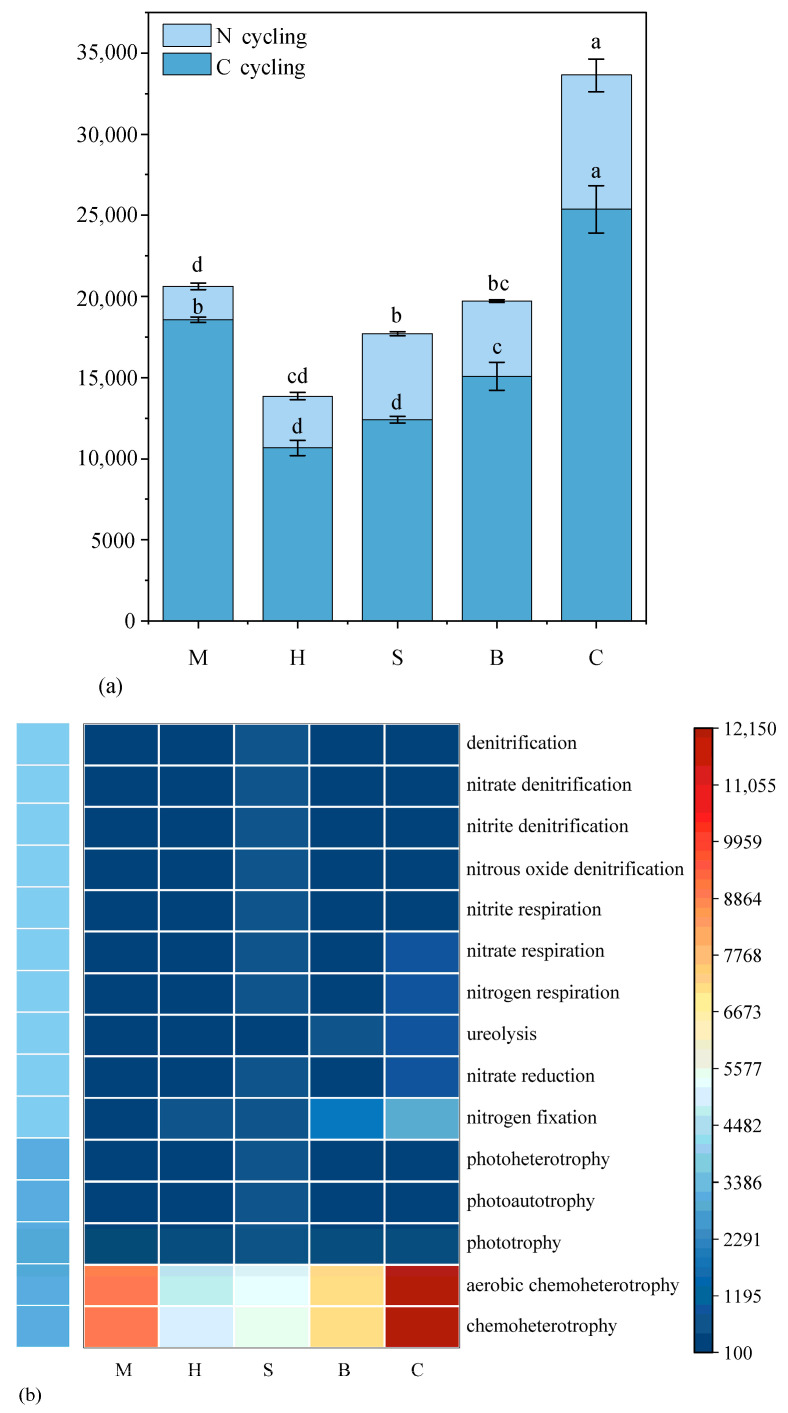
Soil bacterial functional groups with mean relative abundance > 1%. Values are expressed as mean ± standard error (*n* = 3). Different lowercase letters within the same row indicate significant differences among vegetation types at the 0.05 level. M: moss; H: herb; S: shrub; B: broadleaf forest; C: coniferous and broad-leaved mixed forest. (**a**): Abundance of functional populations related to soil carbon and nitrogen cycling across different vegetation types; (**b**): Abundance of functional populations associated with carbon and nitrogen cycling across different vegetation types.

The soil bacterial function was predicted and annotated by FAPROTAX. Fifteen bacterial functional groups exhibited relative abundances greater than 1% (Figure 7a). Bacterial functional groups differed significantly among vegetation types (*p* < 0.05). Chemoheterotrophy and aerobic chemoheterotrophy, as key functional groups in the carbon cycle, exhibited the greatest functional potential in the M group (Table S1). Meanwhile, nitrogen fixation, as another major functional group, showed the highest value in the C group. Functional groups associated with the nitrogen cycle, including nitrate reduction, ureolysis, nitrogen respiration, nitrate respiration, phototrophy, photoautotrophy, nitrite respiration, nitrous oxide denitrification, nitrite denitrification, nitrate denitrification and denitrification, exhibiting the greatest value in S (Figure 7b).

## 4. Discussion

### 4.1. Soil Physical and Chemical Properties in Different Vegetation Types

Soil physicochemical properties were affected by different vegetation types by shifting the amount and composition of litter input. Our research showed that soils in Shrubs had the highest pH value and concentrations of MC, TOC, TN, AP, AN, NN, while the Moss area showed the lowest values. By resetting biological succession, volcanic eruptions produce initial soils that are oligotrophic and depleted in total nitrogen and organic carbon [31]. As the eruption took place only three centuries ago, the primary succession starts from bare rocks, the moss community is the initial stage of succession, and the nutrient content of the soil is low [32]. The Shrubs community tends to thrive in rock crevices and gullies, where microsites with concave surfaces and coarse substrates promote the accumulation of soil, water, and litter [33]. This phenomenon likely accounts for the elevated soil nutrient content observed in shrub communities.

### 4.2. Diversity and Structures of Bacterial Communities in Different Vegetation Types

In this volcanic habitat, herb and shrub communities (S) exhibited significantly higher soil bacterial alpha diversity—reflected by the Ace and Shannon indices—than moss (M) and broadleaf forest (B) communities. The moss community soil (M) is in the early stage of pedogenesis, where lava weathering has just begun. The soil layer is extremely thin, organic matter is severely depleted, and environmental stress is intense, thus supporting only a few stress-tolerant bacterial taxa [34]. Although the broad-leaved forest community soil (B) is rich in organic matter, substantial litter accumulation has led to soil acidification, and the decrease in pH restricts bacterial groups that prefer neutral environments [35]. Consequently, the shrub community soil exhibits the highest bacterial alpha diversity along the lava platform soil development sequence. Our results underscore the critical role of soil attributes in driving bacterial diversity, implying that comparable vegetation–soil–microbe interactions likely exist in other volcanic environments [36]. Shrub communities exhibited higher alpha diversity as a direct biological response to the improved soil conditions they generated [37]. Bacterial community differences among different vegetation types were significantly greater than those within the same vegetation type, indicating that vegetation type is a key driver of beta diversity [38]. Through pathways such as litter quality, root exudates, and root structure, vegetation shapes differentiated soil microhabitats, consequently leading to bacterial community differentiation [39].

Microbial communities are strongly influenced by vegetation type, as it has soil conditions and species-specific litter and root exudates [40,41]. Proteobacteria, Acidobacteria, and Actinobacteria—widely reported as dominant phyla in oligotrophic or disturbed soils—were also prevalent in our study, consistent with the nutrient-poor conditions typical of volcanic lava environments [42]. Actinobacteria and Proteobacteria are commonly associated with early succession stages and are key inhabitants of basaltic substrates, contributing significantly to rock weathering, organic matter decomposition, and nutrient recycling [43,44]. Proteobacteria gradually increases with vegetation succession. The phylum belongs to the eutrophic type, the faster growing eutrophic bacteria have an advantage over the poorly growing bacteria, allowing the eutrophic group to utilize more unstable carbon [45].

Microbial community structure exhibits significant variability at the family level. In different vegetation types, *Xanthobacteraceae* and *norank_o__Vicinamibacterales* were the dominating family. Many studies showed that *Xanthobacteraceae* can disturb important ecological processes such as the decomposition of organic matter and nitrogen cycling in their habitat [46]. Notably, *Xanthobacteraceae* abundance tends to increase gradually during succession sequences. Following a volcanic eruption, nitrogen deficiency is observed on the lava plateau; however, as succession advances, the accumulation of litter mitigates nitrogen deficiency. *Pseudonocardia* species are known to exhibit high UV radiation resistance [47]. Our research has identified the highest prevalence of *Pseudonocardia* in moss. Moss, acting as a pioneer species in the succession process, thrives extensively on barren sandy areas exposed to intense ultraviolet radiation and arid conditions, the same result has been found in the Central Andes [48].

### 4.3. Relationships Between Soil Bacteria and Soil Properties

Microbial colonization in soil is influenced by pH, organic matter, vegetation, and moisture content [49]. There is a limited pH range that is ideal for bacterial development; pH directly affects the status, transformation, and availability of soil nutrients, indirectly shaping microbial communities [35,36]. This study found that pH, moisture content (MC), and total phosphorus (TP) were key factors driving variation in soil microbial community structure. Since reduced soil water content influences microbial metabolic activity and indirectly alters microbial metabolic activity by influencing plant development and nutrient cycling, soil MC was the most significant factor controlling the makeup of the fungal community [36]. As a key environmental factor, soil pH shapes bacterial communities through direct physiological effects—such as limiting proton pump function and protein integrity—and indirect effects mediated by changes in nutrient availability [50]. Thus, different vegetation types selectively assembled distinct microbial communities by modulating these core soil parameters [51]. These differences may be the result of a combination of the soil properties and vegetation characteristics.

In WDLC, the Acidobacteria showed significant positive associations with soil pH and moisture content (MC), but a significant negative relationship with total phosphorus (TP), with its relative abundance being highest in nutrient-poor Moss communities. Acidobacteria and Actinobacteria are considered oligotrophic bacteria that thrive in nutrient-limited conditions and outcompete other taxa in resource-poor soils [52]. Acidobacteria exhibited a significant positive association with both soil pH and moisture content (MC), and significantly negatively correlated with total phosphorus (TP), with its relative abundance being highest in nutrient-poor Moss communities. In contrast, Actinobacteria shows the opposite pattern, indicating that although both belong to oligotrophic bacteria, there is competition between them even within the same habitat [53].

### 4.4. Potential Functional Group of Soil Microbial

The soil bacterial functional groups play a crucial role in biogeochemical cycling; soil and vegetation types may exert stronger influences on microbial functions [54]. In our study, the dominant functional group was chemoheterotrophy and aerobic chemoheterotrophy in different vegetation types, showing that the majority of the microbes cannot fix carbon and must derive carbon and energy through the oxidation of organic compounds [55,56]. The high levels of chemoheterotrophy and aerobic chemoheterotrophy in moss facilitate the decomposition of organic matter, thereby enhancing soil nutrient availability to support the nutrient requirements essential for plant succession [57]. Among the key soil properties, moisture content (MC), pH, and total phosphorus (TP) have been identified as critical determinants shaping bacterial community structure and function in volcanic ecosystems.

The nitrogen cycling functional groups of the soil of different Vegetation Types in volcanic Lava are mainly nitrogen fixation, nitrate reduction, ureolysis, nitrogen respiration, nitrate respiration. Nitrogen fixation was also a major functional group, exhibiting the greatest value in B and C. Members of the family Nitrobacteraceae are key contributors to nitrogen cycling by participating in nitrogen fixation and the transformation of organic nitrogen [58]. Importantly, these functional groups are strongly shaped by key soil factors (MC, pH, TP) that determine both bacterial community structure and nitrogen-cycling function [59]. In our lava soils, near-neutral pH in herb and shrub communities supports more diverse and functionally rich nitrogen-transforming assemblages, whereas more acidic forest soils tend to favor specialized groups such as low-pH-adapted nitrogen-fixing bacteria [60]. Soil moisture content (MC) regulates oxygen availability and thus the balance between aerobic processes (e.g., nitrification, aerobic nitrate reduction) and anaerobic nitrogen respiration, thereby directly influencing the relative contributions of different nitrogen-cycling pathways [61]. In addition, variation in total phosphorus (TP) across vegetation types alters nutrient stoichiometry and microbial nutrient limitation, which can further modulate the abundance of key nitrogen-cycling taxa and functional genes, especially in nutrient-poor volcanic substrates [62].

## 5. Conclusions

The emergence of diverse plant communities after volcanic activity creates a valuable context for investigating how soil microbial communities assemble and functional adaptation ecologically significant. Across these evolving ecosystems, soil microbial diversity, community composition, and functional potential are predominantly shaped by key soil physicochemical properties. Wudalianchi Lava Plateau soil moisture content (MC), pH, and total phosphorus (TP) are the primary environmental drivers influencing bacterial community structure. Shrub vegetation enhances soil nutrient availability, modifies the soil microhabitat, and fosters a distinct microbial community. Notably, microbial functional groups associated with carbon metabolism, such as chemoheterotrophy and aerobic chemoheterotrophy, were significantly enriched in moss, indicating a vegetation-specific prioritization of key ecological processes. This study establishes a linkage framework connecting post-eruption vegetation succession, soil physicochemical gradients, and the structure and function of soil microbial communities, providing insights into ecosystem recovery mechanisms in volcanic environments. However, the current findings are constrained by the spatial and temporal scope of sampling—samples were collected only once from a limited number of sites—which may not fully capture the dynamic variability of post-volcanic ecosystems. Therefore, these results should be interpreted with caution, and further validation is needed to establish causal relationships between specific vegetation traits and microbial functional outcomes. The current findings, constrained by the spatial and temporal scope of sampling, highlight the need for further validation of the causal relationships between specific vegetation traits and microbial functional outcomes. Future research should further refine the proposed “vegetation–soil–microbe” dynamic model by expanding spatiotemporal sampling and by conducting comparisons across different volcanic systems.

## Figures and Tables

**Table 1:** Overview of the study sites.

Vegetation Type	Soil Type	Dominant Species	Coverage (%)	Altitude (m)
Moss (M)	Raw volcanic ash soil	*Racomitrium canescens*	70–90	332–334
Herb (H)	Black volcanic ash soil	*Potentilla chinensis*, *Artemisia sacrorum*, *Patrinia rupestris*	70–80	327–348
Shrub (S)	Black volcanic ash soil	*Sorbaria sorbifolia*, *Rubus sachalinensis*	20–40	328–354
Broadleaf forest (B)	Dark brown volcanic soil	*Populus davidiana*, *Betula platyphylla* and *P*. *koreana*	85–90	329–350
Mixed coniferous-broad-leaved forest (C)	Dark brown volcanic soil	*Betula platyphylla*, *Larix gmelinii*	65–80	320–345

**Table 2:** Soil physical and chemical characteristics of different vegetation types (M: moss; H: herb; S: shrub; B: broadleaf forest; C: coniferous and broad-leaved mixed forest) in volcanic lava. Abbreviations: MC (moisture content), TOC (total organic carbon), TN (total nitrogen), TP (total phosphorus), AP (available phosphorus), AN (ammonium nitrogen), NN (nitrate nitrogen). The data in the table represent mean ± standard error (*n* = 3). Different lowercase letters in the same column represent significant differences (*p* < 0.05).

Sample	pH	MC (%)	TOC (g·kg^−1^)	TN (g·kg^−1^)	TP (g·kg^−1^)	AP (mg·kg^−1^)	AN (mg·kg^−1^)	NN (mg·kg^−1^)
M	6.40 ± 0.06 ^d^	13.23 ± 0.26 ^c^	6.69 ± 0.33 ^e^	0.37 ± 0.04 ^d^	3.30 ± 0.64 ^a^	12.16 ± 2.21 ^c^	3.90 ± 0.29 ^d^	2.60 ± 0.31 ^b^
H	6.83 ± 0.04 ^b^	19.26 ± 0.19 ^b^	24.29 ± 0.67 ^d^	2.03 ± 0.06 ^c^	1.63 ± 0.18 ^c^	11.01 ± 1.04 ^c^	5.15 ± 0.42 ^c^	3.39 ± 0.08 ^b^
S	6.98 ± 0.04 ^a^	43.59 ± 0.36 ^a^	94.75 ± 4.9 ^a^	5.68 ± 0.28 ^a^	2.45 ± 0.10 ^b^	21.01 ± 2.03 ^a^	8.88 ± 0.31 ^a^	11.52 ± 1.74 ^a^
B	6.56 ± 0.01 ^c^	31.28 ± 1.8 ^ab^	48.24 ± 1.46 ^b^	2.84 ± 0.05 ^b^	2.21 ± 0.37 ^bc^	13.84 ± 1.6 ^bc^	6.11 ± 0.13 ^b^	2.99 ± 0.03 ^b^
C	6.62 ± 0.01 ^c^	20.20 ± 0.78 ^b^	38.57 ± 0.32 ^c^	1.82 ± 0.02 ^c^	1.93 ± 0.22 ^bc^	16.12 ± 0.23 ^b^	4.83 ± 0.32 ^c^	2.99 ± 0.15 ^b^

**Table 3:** Soil bacterial diversity indices for each vegetation type (M: moss; H: herb; S: shrub; B: broadleaf forest; C: coniferous and broad-leaved mixed forest). Data are presented as mean ± standard error (*n* = 3). Significant differences among vegetation types (*p* < 0.05) are denoted by different lowercase letters within the same column, based on one-way ANOVA with Duncan’s post hoc test. The absence of letters indicates no significant differences among groups.

Sample	Ace	Shannon	Coverage	Simpson	Pd
M	2311.37 ± 77.36 ^c^	6.44 ± 0.04 ^c^	0.9982 ± 0.0008 ^a^	0.02 ± 0.00 ^d^	211.83 ± 9.46 ^b^
H	3997.64 ± 181.90 ^a^	7.50 ± 0.03 ^a^	0.9909 ± 0.0008 ^c^	0.22 ± 0.01 ^a^	428.83 ± 26.02 ^a^
S	3626.78 ± 223.57 ^a^	7.41 ± 0.05 ^a^	0.9928 ± 0.0002 ^bc^	0.23 ± 0.01 ^a^	383.15 ± 35.51 ^a^
B	3124.32 ± 127.99 ^b^	7.10 ± 0.07 ^b^	0.9961 ± 0.0020 ^ab^	0.14 ± 0.02 ^b^	360.24 ± 6.38 ^a^
C	2149.81 ± 71.98 ^c^	6.50 ± 0.05 ^c^	0.9962 ± 0.0014 ^ab^	0.10 ± 0.01 ^c^	231.13 ± 19.08 ^b^

## Data Availability

The original contributions presented in this study are included in the article. Further inquiries can be directed to the corresponding authors. The raw reads were deposited into the NCBI Sequence Read Archive (SRA) database Bacterial Accession Number PRJNA1356813.

## Supplementary Materials

The following supporting information can be downloaded at: https://www.mdpi.com/article/10.3390/microorganisms14030666/s1, Table S1: Differences in potential functional groups of soil bacteria with average relative abundance >1%. The data in the table are mean ± standard error (*n* = 3). If the letter is the same, the divergence is not significant; on the contrary, the divergence is significant at the 0.05 level. M: moss; H: herb; S: shrub; B: broadleaf forest; C: coniferous and broad-leaved mixed forest.
