# Supplementary material for: Diversity and Community Structure of Soil Bacteria of Different Vegetation Types in Volcanic Lava of Wudalianchi, China
Source: Microorganisms. 2026 Mar 15;14(3):666. doi: 10.3390/microorganisms14030666 (PMC13029127; doi:10.3390/microorganisms14030666)
Supplement: Supplementary file 1 [file microorganisms-14-00666-s001.zip › microorganisms-4170151-supplementary.pdf]

Supplementary Table S1. Differences in potential functional groups of soil bacteria with average relative abundance >1%. The data in the table are mean ± standard error (n = 3). If the letter is the same, the divergence is not significant; on the contrary, the divergence is significant at the 0.05 level. M: moss; H: herb; S: shrub; B: broadleaf forest; C: coniferous and broad-leaved mixed forest.

	Sample	M (%)	H (%)	S (%)	B (%)	C (%)
C cycling	Chemoheterotrophy	43.5±0.72 a	35.14±0.29 b	30.68±0.42 c	35.93±0.59 b	36.06±0.59 b
	aerobic chemoheterotrophy	43.29±0.68 a	34.55±0.31 b	29.92±0.37 c	35.72±0.6 b	35.76±0.62 b
	phototrophy	1.15±0.14 d	2.65±0.1 b	3.32±0.08 a	1.7±0.16 c	1.31±0.14 cd
	photoautotrophy	1.14±0.13 d	2.43±0.07 b	3.18±0.1 a	1.59±0.16 c	1.23±0.15 cd
	photoheterotrophy	0.89±0.12 d	2.23±0.07 b	3.01±0.09 a	1.44±0.19 c	1.24±0.13 cd
N cycling	nitrogen fixation	2.09±0.23 b	4.04±0.23 b	3.43±0.07 b	10.12±0.92 a	8.74±1.47 a
	nitrate reduction	1.19±0.04 c	2.91±0.23 ab	3.48±0.14 a	1.68±0.22 bc	2.44±1.06 abc
	ureolysis	0.55±0.10 c	1.64±0.09 b	2.49±0.32 a	2.44±0.23 a	2.66±0.35 a
	nitrogen respiration	0.91±0.13 b	2.15±0.06 ab	3.03±0.12 a	1.39±0.2 ab	2.36±1.08 ab
	nitrate respiration	0.91±0.13 b	2.15±0.06 ab	3.03±0.12 a	1.39±0.2 ab	2.36±1.08 ab
	nitrite respiration	0.88±0.11 d	2.02±0.06 b	2.9±0.11 a	1.32±0.18 c	1.17±0.14 cd
	nitrous oxide denitrification	0.88±0.11 d	2.02±0.06 b	2.88±0.11 a	1.32±0.18 c	1.17±0.14 cd
	nitrite denitrification	0.88±0.11 d	2.02±0.06 b	2.88±0.11 a	1.32±0.18 c	1.17±0.14 cd
	nitrate denitrification	0.88±0.11 d	2.02±0.06 b	2.88±0.11 a	1.32±0.18 c	1.17±0.14 cd
	denitrification	0.88±0.11 d	2.02±0.06 b	2.88±0.11 a	1.32±0.18 c	1.17±0.14 cd
